# From GWAS to Gene: Transcriptome-Wide Association Studies and Other Methods to Functionally Understand GWAS Discoveries

**DOI:** 10.3389/fgene.2021.713230

**Published:** 2021-09-30

**Authors:** Binglan Li, Marylyn D. Ritchie

**Affiliations:** ^1^Department of Biomedical Data Science, Stanford University, Stanford, CA, United States; ^2^Department of Genetics, University of Pennsylvania, Philadelphia, PA, United States; ^3^Institute for Biomedical Informatics, University of Pennsylvania, Philadelphia, PA, United States

**Keywords:** TWAS, GWAS, summary statistics, data integration, eQTL, functional annotation, fine-mapping

## Abstract

Since their inception, genome-wide association studies (GWAS) have identified more than a hundred thousand single nucleotide polymorphism (SNP) loci that are associated with various complex human diseases or traits. The majority of GWAS discoveries are located in non-coding regions of the human genome and have unknown functions. The valley between non-coding GWAS discoveries and downstream affected genes hinders the investigation of complex disease mechanism and the utilization of human genetics for the improvement of clinical care. Meanwhile, advances in high-throughput sequencing technologies reveal important genomic regulatory roles that non-coding regions play in the transcriptional activities of genes. In this review, we focus on data integrative bioinformatics methods that combine GWAS with functional genomics knowledge to identify genetically regulated genes. We categorize and describe two types of data integrative methods. First, we describe fine-mapping methods. Fine-mapping is an exploratory approach that calibrates likely causal variants underneath GWAS signals. Fine-mapping methods connect GWAS signals to potentially causal genes through statistical methods and/or functional annotations. Second, we discuss gene-prioritization methods. These are hypothesis generating approaches that evaluate whether genetic variants regulate genes via certain genetic regulatory mechanisms to influence complex traits, including colocalization, mendelian randomization, and the transcriptome-wide association study (TWAS). TWAS is a gene-based association approach that investigates associations between genetically regulated gene expression and complex diseases or traits. TWAS has gained popularity over the years due to its ability to reduce multiple testing burden in comparison to other variant-based analytic approaches. Multiple types of TWAS methods have been developed with varied methodological designs and biological hypotheses over the past 5 years. We dive into discussions of how TWAS methods differ in many aspects and the challenges that different TWAS methods face. Overall, TWAS is a powerful tool for identifying complex trait-associated genes. With the advent of single-cell sequencing, chromosome conformation capture, gene editing technologies, and multiplexing reporter assays, we are expecting a more comprehensive understanding of genomic regulation and genetically regulated genes underlying complex human diseases and traits in the future.

## Introduction

For the last two decades, genome-wide association studies (GWAS) have been a successful approach for associating single nucleotide polymorphism (SNP) loci to a variety of complex human traits. In fact, as of July 2021, the NHGRI-EBI GWAS catalog includes more than 167,000 SNPs associated with human diseases and traits (Buniello et al., 2019). The abundant discoveries of SNP associations with complex human diseases have led to significant enthusiasm and growth in interdisciplinary, translational medicine studies. Translational medicine aims to translate genomic discoveries of complex human diseases to clinical settings to achieve precision medicine (Collins and Varmus, 2015) and to improve the overall quality of health care. The expedition from bench to bedside investigates genetically determined disease susceptibility and inter-individual variability in treatment response to develop genomics-informed diagnosis and prognosis tools as well as individually tailored treatment plans. However, the majority (∼90%) of statistically significant GWAS signals are located in non-coding regions of the human genome (Maurano et al., 2012). Thus, connecting these non-coding variants to downstream affected genes is a nontrivial task. The gap between non-coding GWAS signals and affected genes hinders the translation of GWAS discoveries to clinical settings.

Increased volume and improved precision of omics data, newly invented molecular technologies, and recently developed bioinformatics algorithms, together reveal novel avenues in translational medicine to walk from GWAS signals to downstream affected genes. Non-coding regions of the human genome, including intergenic and intronic regions, can act as regulatory elements that have effects on transcriptional or translational activities of genes. Several classes of widely-studied functional elements include enhancers, promoters, transcription factor binding sites (TFBS), CCCTC-binding factor (CTCF); and these functional elements can host genetic variants, like expression quantitative trait loci (eQTLs), splicing quantitative trait loci (sQTLs), and protein quantitative trait loci (pQTLs), which participate in various transcriptional and translational regulatory mechanisms [Visel et al., 2007; The FANTOM Consortium and the RIKEN PMI and CLST (DGT), 2014; Andersson et al., 2014; Roadmap Epigenomics Consortium et al., 2015; Sun et al., 2018; ENCODE Project Consortium et al., 2020; GTEx Consortium, 2020]. Each class of functional element describes a type of regulatory mechanism by which genetic variants may modulate genes. The goals of many developed bioinformatics methods in the post-GWAS era are to identify genetically regulated genes from GWAS discoveries by integrating functional genomics knowledge. Transcriptome-wide association studies (TWAS) are one type of data integrative bioinformatics method that aims to identify genes that lead to manifestation of complex human traits due to genetically regulated transcriptional activity.

Transcriptome-wide association studies has gained popularity over the years due to its distinct ability to perform gene-level association analyses and generate interpretable transcription hypotheses between genes and complex diseases and traits. Here, we first review updates in functional genomics. We also summarize bioinformatics methods that embrace functional genomics data to identify complex trait-associated genes. Then, we dive into the specifics of TWAS and assess the pros and cons of several developed TWAS methods. Next, we discuss several influential factors in the experimental design of TWAS that may potentially sway interpretation of results. Finally, we review challenges for TWAS and opportunities to maximize the utility of TWAS in the future.

## Overview

The technological advances to identify genomic regulation provide opportunities to prioritize genetically regulated genes from GWAS signals from new perspectives. Fine-mapping of GWAS causal signals has relied heavily on linkage disequilibrium (LD). A common practice following GWAS is to map genetic variants to the residing genes, or nearby genes based on haplotypes and LD structures derived from the study cohort or from a fully sequenced reference panel of presumably similar ancestry [such as an ancestrally similar subset of the 1000 Genomes Project (1000 Genomes Project Consortium et al., 2015)]. This approach has led to identification of complex disease and trait-associated loci, but does not recognize the widespread, complex transcriptional regulatory mechanisms which do not necessarily take place in genes’ proximity (Heidari et al., 2014; Javierre et al., 2016; Pan et al., 2018).

**FIGURE 1:**
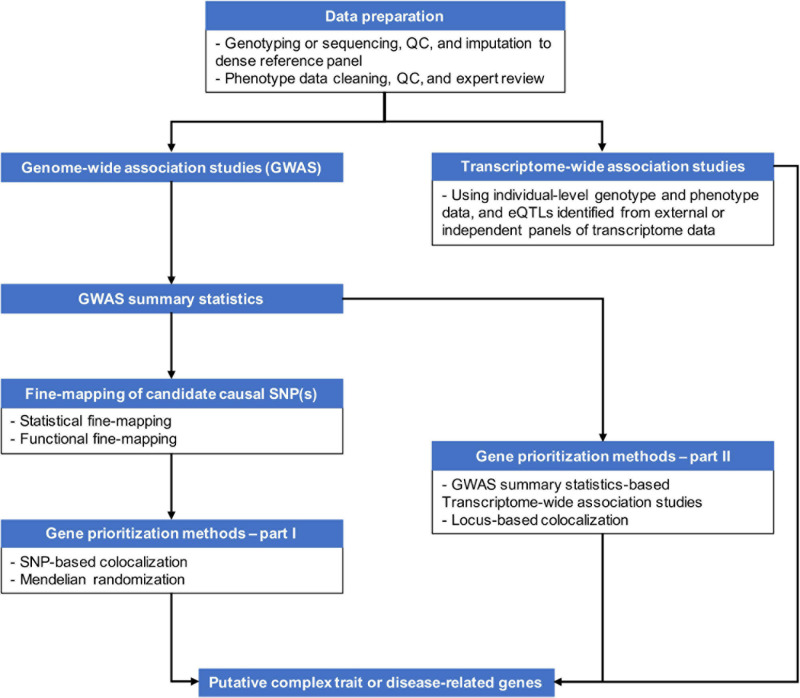
An overview of strategies for gene-mapping following GWAS or parallel to GWAS.

Genetic variants, regardless of their chromosome locations relevant to genes, can modulate transcriptional activities of target genes up to several mega base pairs (Mbp) away if located in regulatory elements, such as enhancers and transcriptional factor (TF) binding sites, or having suggestive effects on genes, like expression quantitative trait loci (eQTLs) (Javierre et al., 2016; GTEx Consortium, 2020). The distal genomic regulations are accomplished via formations of chromatin loops. As more knowledge about three-dimensional (3D) genome structure becomes available through chromosome conformation capture (3C) technology and its derivatives (Davies et al., 2017), it becomes well-recognized that chromatin looping plays an important role in controlling transcriptional activities (Dixon et al., 2012; Rao et al., 2014). Chromatin looping allows distal regulatory elements to skip intervening genes to contact distant target genes. For example, using the 3C-carbon copy (5C) approach, Sanyal et al. (2012) observed that only ∼7% of chromatin looping interactions took place between an element (putative enhancers, promotors or CTCF binding sites) and the nearest transcription start site (TSS) in the pilot regions that represented 1% of the human genome in GM12878, K562, and HeLa-S3 cell lines (ENCODE Project Consortium, 2012). Even though Sanyal et al. (2012) inspected only a small proportion of the human genome, the frequency of distal regulatory interactions is profound. Proximity to genes or short-range *cis-*LD structures may not be sufficient tools to pinpoint causal genes of complex traits and diseases given the continuously updating knowledge of genomic regulation. Integration of genetic regulatory knowledge with GWAS results has become necessary to capture the complexity of biological regulatory mechanisms and prioritize genes from GWAS signals. Figure 1 provides an overview of some of the strategies for post-GWAS gene-mapping procedures.

As of today, there have been various statistical and computational methods that incorporate functional genomics data to unveil complex trait-related genes. In this review, we categorize these methods into two types. First we describe the fine-mapping approach. Second we discuss the gene-prioritization approach.

### Fine-Mapping for Post-GWAS Analysis

Fine-mapping is one common option for post-GWAS analyses seeking to identify causal variants or genes for complex diseases or traits (Schaid et al., 2018; Broekema et al., 2020). Traditionally, fine-mapping of potential causal variants relies heavily on LD structures and haplotypes blocks based on the premise that causal variants and tag variants have a non-random chance to be inherited together due to co-segregation during meiotic recombination (Table 1). Recently, there have also been multiple studies on alternative functional fine-mapping strategies that aim to identify potential causal functional elements, instead of a single variant, tagged by GWAS signals. These functional fine-mapping studies investigate downstream affected genes by understanding the likely impacted biological regulatory mechanisms. This shift of focus in GWAS fine-mapping is transformative for studies which are perplexed by non-coding GWAS signals and their connections to downstream affected genes (Table 1).

**TABLE 1:**
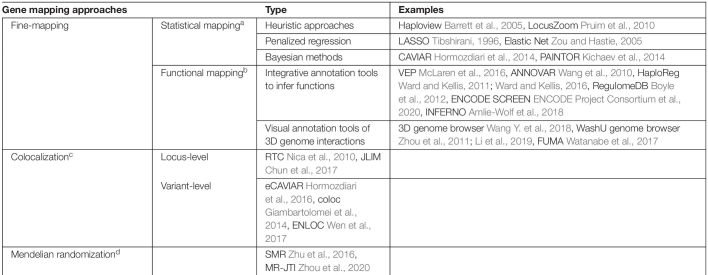
Toolbox of gene-mapping methods and gene-prioritization methods (see Table 2 for TWAS).

*^*a*^ For detailed review of statistical fine-mapping, see Schaid et al. (2018); ^*b*^ For detailed review of functional fine-mapping, see Broekema et al. (2020); ^*c*^ See Hukku et al. (2021) for a detailed review of colocalization methods; ^*d*^ See Davies et al. (2018) for practical guidelines for clinical implementation of MR.*

**TABLE 2:**
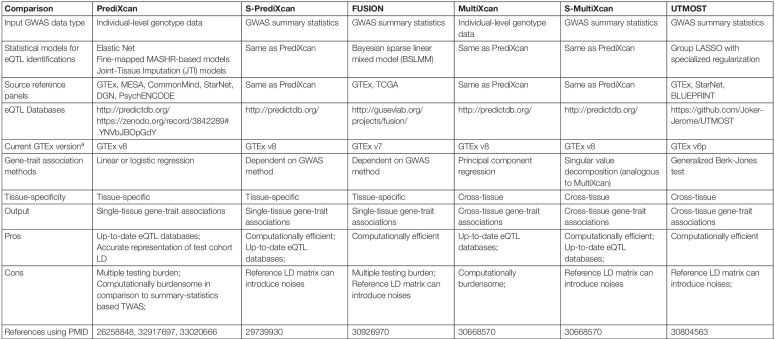
Summary of TWAS methods.

*^*a*^ Dated August 2021.*

Fine-mapped GWAS signals may occur outside of coding regions and be situated in a distant non-coding functional element. Identification of non-coding causal functional elements is imperative for understanding the functional roles of GWAS variants. Examples of non-coding functional roles are enhancers, promoters, TF binding sites, candidate *cis-*regulatory elements (ccREs), and DNaseI hypersensitive sites. The identification of functional elements underlying GWAS pave the way to engage chromosome conformation information to locate the downstream target genes interacting with the functional regions of interest. The Washington Epigenome Browser (Zhou et al., 2011; Li et al., 2019) and 3D genome browser (Wang Y. et al., 2018) host several different kinds of cell line-specific or tissue-specific 3C, 5C, Hi-C, or capture Hi-C data. Both browsers provide necessary visualization tools to inspect the 3D chromatin loop-aided interactions for genomic regions of interest. FUMA developed by Watanabe et al. (2017) is another data integrative computational tool to assist functional annotation of fine-mapped GWAS variants and functional regions. Watanabe et al. (2017) assembles positional, eQTL, and chromosome confirmation mappings in FUMA. FUMA offers interactive visual aids for post-GWAS functional annotation and prioritization of potential complex trait-related genes based on multiple types of functional genomics data.

Table 1 lists exemplary methods of two major types of fine-mapping approaches. The statistical mapping focuses on the statistical approaches and models. The functional mapping focuses on the varied ways of using different functional genomic data for fine-mapping purposes. These two types of fine-mapping approaches are not mutually exclusive. A fine-mapping method can also fall into both categories depending on the method or study design. To summarize, fine-mapping methods integrate various types of omics data to deduct possible variant-gene relationships and biological mechanisms underpinning complex diseases or traits.

### Gene-Prioritization for Post-GWAS Analysis

The capability of high-throughput sequencing technologies to quantify intermediate molecular traits, such as gene expression levels and protein abundance, enables the estimation of statistical significance of molecular mechanisms behind complex diseases and traits. Here, we discuss three different types of gene-prioritization methods that to evaluate how genetic variants can modify complex disease risk by exerting effects on an intermediate molecular trait.

One such integrative gene-prioritization method is colocalization (Table 1; Hukku et al., 2021). In general, colocalization analyzes the co-occurring patterns between QTLs (for example, eQTLs) and GWAS signals. Colocalization assesses the biological hypothesis of whether a causal locus or a genetic variant contribute to both the intermediate molecular changes and the complex trait of interest. A GWAS signal that is colocalized with a QTL is more likely to be functional. Colocalization analyses can be performed at a locus level or at a SNP level.

The locus-level colocalization methods assume that a group of SNPs in a tight LD region contain both a causal eQTL and a causal disease GWAS signal (Table 1). One will observe no marginal effect of a causal eQTL by conditioning on the most significant disease GWAS signal, and vice versa (Nica et al., 2010). An alternative method states that one will observe a maximum joint likelihood of associations if the two traits of interest are driven by the same causal variant (Chun et al., 2017).

The SNP-level colocalization methods focus on quantifying the probability of colocalization signals of two distinct traits surrounding a suspected causal variant (hence, at the single SNP/variant resolution) (Table 1). Several exemplary SNP-level colocalization methods include eCAVIAR (Hormozdiari et al., 2016), COLOC (Giambartolomei et al., 2014), ENLOC (Wen et al., 2017), and fastENLOC (Pividori et al., 2020).

Mendelian Randomization (MR) is another approach, which makes causal inference between a modifiable exposure and complex disease risk (Holmes et al., 2017). The modifiable exposure can be blood concentrations of low-density lipoprotein cholesterol (LDL-c). The complex disease can be coronary heart disease (CHD). LDL-c related genetic variants are used in the process as instrumental variables to estimate the causal effects of LDL-c on CHD risk. One rising MR approach harnesses eQTLs to investigate whether one or more genetic variants influence both gene expression and a complex trait at the same time. This approach estimates, for example, if a *PCSK9* eQTL regulates *PCSK9* gene expression levels to impact blood LDL-c levels (Taylor et al., 2019; Richardson et al., 2020). eQTL-instrumented MR analyses are an innovative means to investigate LDL-related genes, which may further contribute to CHD risk. However, the success and accurate interpretation of MR results depend on three key assumptions (Holmes et al., 2017; Davies et al., 2018). Following the *PCSK9* eQTL and LDL-c example: (1) the genetic variant must be associated with gene expression levels; (2) there cannot be unmeasured confounding effects between the genetic variant and LDL-c; and (3) the genetic variant affects LDL-c only through their effects on gene expression levels.

Transcriptome-wide association study is a gene-based association approach first developed by Gamazon et al. (2015). TWAS integrates GWAS data with eQTL information to identify transcriptionally regulated genes underlying complex traits and diseases. TWAS first imputes the genetically regulated gene expression levels by combining individual-level genotype data or GWAS summary statistics with externally estimated eQTLs. At the second step, TWAS assesses the associations between imputed gene expression levels and a complex trait or disease (see section “Introduction to TWAS”).

Transcriptome-wide association studies and mendelian randomization are similar in the way that TWAS is equivalent to a two-stage weighted allele score-based MR. The first stage estimates the aggregate effect of multiple instrumental variables on the exposure (for example, eQTLs’ aggregate effect on a gene). The second stage regresses the outcome on the fitted values of the exposure from the first stage (for example, regression of continuous or categorial disease-related phenotype on the predicted genetically regulated gene expression levels). More interdisciplinary details can be found in Burgess et al. (2017); Burgess and Thompson (2013), and Pierce and Burgess (2013). The rest of this review focuses on the statistical aspects of TWAS as a gene-based association approach.

Transcriptome-wide association studies have attracted much interest in the field of complex disease due to its ability to perform gene-level association testing. This feature distinguishes TWAS from variant-based analytic approaches, such as some of the aforementioned fine-mapping, colocalization, or MR. These variant-based analytic approaches rely greatly on GWAS ability to identify complex trait or disease-related genetic variants. However, detecting variants with small to moderate effects requires considerable sample sizes in order to reach satisfactory statistical power (McCarthy et al., 2008; Manolio et al., 2009). TWAS overcomes this issue by aggregating regulatory effects of multiple eQTLs and directly testing associations between genes and diseases. Moreover, TWAS has a substantially smaller multiple testing burden by performing gene-level tests in comparison with variant-based analyses. Furthermore, TWAS is a flexible bioinformatics tool. TWAS can be used as an accessory to GWAS to support GWAS discoveries; or independently from GWAS (Figure 1). Some studies include TWAS as a parallel approach to their GWAS to identify putative causal genes associated with complex disease risk. The following sections focus on the variations of TWAS methods and the influential factors of TWAS studies.

## Transcriptome-Wide Association Studies (TWAS)

### Introduction to TWAS

Transcriptome-wide association studies can be considered a subclass of locus-based methods or multi-marker association approaches that are an alternative to variant-based association methods. The growth of locus-based methods is attributable to the wider recognition and appreciation of the polygenic architecture of complex diseases and traits. In other words, the proportion of disease phenotype variation explained by each genetic variant, on average, is small. Nevertheless, the cumulative effect of genetic variants in many genes, collectively, account for a substantial proportion of inter-individual phenotypic variation. Methodologically, locus-based methods take multiple genetic variants’ effects into account to assess the overall contribution of a gene or a genetic region (a more interpretable functional unit in comparison to non-coding variants) to complex disease susceptibility. Meanwhile, advances in high-throughput sequencing technologies have enriched the discovery that genetic variants are tightly involved in regulation of transcription and translation of genetic material. eQTLs are one type of important regulatory variants. Recently, the detection of eQTLs has been aided by even lower cost RNA sequencing (RNA-seq) technology, sophisticated statistical models, increasing computational power, and scientific community efforts to consolidate eQTL research resources.

Similar to the shift in the GWAS field from variants with large effect sizes to variants with moderate to small effect sizes by involving greater sample sizes, eQTL research has gone through the same trend. eQTLs with large effect have elucidated molecular mechanisms behind a variety of complex diseases. For example, a promoter eQTL has a dominant genetic effect on *DARC*, a gene expressing malaria parasite receptor. The specific form of the eQTL interrupts *GATA*-1 binding sites and diminishes *DARC* gene expression in specific erythroid cells, which explains malaria resistance found in a certain West African population (Tournamille et al., 1995). Examples like the *DARC* promoter eQTL with a silencing effect are not common. The ability of the community to assemble even larger study cohorts allows for the observations of additional eQTLs, albeit with smaller effect sizes, and in a diverse pool of tissue types other than blood. TWAS adopts this polygenic view including multiple small effect eQTLs for exploring the genetic architecture of complex disease risk.

Transcriptome-wide association studies exploit the genotype and phenotype data from GWAS along with reference transcriptome data to conduct gene-level association testing (Gamazon et al., 2015; Gusev et al., 2016; Barbeira et al., 2018, 2019; Hu et al., 2019; Pividori et al., 2020). TWAS tests the hypothesis that one or multiple eQTLs collectively regulate the transcriptional activities of a gene, and the genetically altered gene expression levels result in modulated disease risk.

Provided individual-level genome-wide genotype data, TWAS performs a two-step analysis to test this transcriptional hypothesis. For any given gene, step 1 imputes genetically regulated gene expression levels by combining transcriptional regulatory effects of the eQTLs for a gene under an additive genetic model. Step 1 can be done in multiple tissues of interest separately in each tissue or jointly across tissues (see section about “eQTL Detection”). Various eQTL models are available for step 1 thanks to the efforts of consortia, like GTEx (GTEx Consortium, 2020), BLUEPRINT (Chen et al., 2016), eQTLGEN (Võsa et al., 2018), and MESA (Mogil et al., 2018). Let *N* denote the sample size of a study cohort and M denote the number of eQTLs in a certain gene. Prediction of the gene’s genetically regulated gene expression levels can be expressed as follows:


(1) $$E=X\,\hat{W}$$

where *E* is the *N*× 1 vector of predicted genetically regulated gene expression levels of the gene, *X* is the *N*× *M* matrix of genotypes of eQTLs, and $\hat{W}$ is the *M*× 1 vector of eQTLs’ regulatory effects on the gene, which are estimated from an independent reference transcriptome data panel. While the first step of TWAS is merely to capture genetic components of gene expression levels, TWAS has shown to have a good prediction accuracy for genes that are highly locally heritable (*h*^2^ ≥ 0.5) (Gamazon et al., 2015; Li et al., 2018).

The second step is to aggregate the imputed gene expression levels from step 1 with a disease phenotype of interest to estimate the statistical significance of each gene-disease association. Let *Y* denote the phenotype of a study cohort. *Y* is the *N*× 1 vector of phenotype, which can be dichotomous, such as case/control status of a complex disease, or continuous measures of health outcomes, such as blood laboratory values. Step 2 calculates the regression coefficient of the phenotype *Y* on each genes’ predicted gene expression levels *E*, Given its design, TWAS conducts genomic association analyses with an innate transcriptional regulatory hypothesis.

Transcriptome-wide association studies have several advantages over traditional variant-based genomic analyses. First, TWAS is a gene-based analytic approach that has the potential to extend GWAS toward a functional understanding of disease mechanisms. Second, the two analytic steps in TWAS are decoupled and can be conducted independently. For multi-trait or phenome-wide studies, the first step of predicting gene expression levels only needs to be performed once for a given dataset. Predicted genetically regulated gene expression levels can be then evaluated for statistical association with different disease phenotypes or complex traits at step 2. Meanwhile, the technical independence of step 2 gives ample research opportunities for the development of sophisticated statistical models for gene-disease association analyses. Third, multiple testing burden is lower in TWAS in comparison to a genome-wide variant-based test; here, one only needs to adjust for the number of genes tested in the TWAS. For a given trait, a TWAS only needs to adjust for approximately twenty-thousand genes (this is a Bonferroni *p*-value threshold of approximately 2.5× 10^−6^). Meanwhile, the number of statistical tests goes up to millions for a GWAS. As such, the multiple testing burden is orders of magnitude heavier in GWAS than in TWAS. The lower multiple testing burden allowed Thériault et al. (2018) to identify the association between *PALMD* and calcific aortic valve stenosis (CAVS) in the QUEBEC-CAVS cohort with a sample size of *N* = 2,000). The *PALMD*-CAVS association was successfully replicated in the much larger UK Biobank CAVS GWAS (*N* = 353,000). However, the same association was not statistically significant in the QUEBEC-CAVS GWAS due to the great multiple testing burden relative to the limited GWAS sample size (QUEBEC-CAVS *N* = 2,000). Fourth, TWAS are tissue-specific. TWAS has the capability to predict tissue-specific genetically regulated gene expression levels and investigate gene-trait associations in disease-related or potentially pathological tissues.

A TWAS study is subject to several influential factors which merit cautious interpretations of results (Table 2). These influential factors include: (1) the nature of input GWAS data, in other words, individual-level genotype and phenotype data versus GWAS summary statistics, (2) the eQTL models, and (3) the association method used to estimate gene-trait associations. In the following sections, we expand on each of these factors.

### Individual-Level Data-Based TWAS Versus GWAS Summary Statistics-Based TWAS

Transcriptome-wide association studies can take different forms of input data types. The first published TWAS method, PrediXcan, developed by Gamazon et al. (2015), accepts individual-level major variant dosages of eQTLs or genotype calls as input. However, individual-level genotype data are not easily obtainable from published GWAS studies for a TWAS follow-up study. As a solution and an alternative TWAS method, FUSION, developed by Gusev et al. (2016), quickly followed the release of PrediXcan. FUSION imputes the regression statistics between the gene expression level of each gene and a trait (hereafter denoted as *z*_*g*_) directly from GWAS summary statistics. Let *Z* denote a vector of standardized SNP-trait effect sizes (z-scores) from a GWAS and only include GWAS SNPs that are also eQTLs in a given eQTL-gene expression model; and Σ denote the covariance matrix among all eQTLs (LD). In FUSION, *z*_*g*_ are imputed as a linear combination of elements of Z with weights $\hat{W}$. When there is no SNP-trait association (no signals), Z∼*N*(0,Σ) and therefore, *z*_*g*_ has a zero mean and variance ${\hat{W}}^{\prime }$Σ$\hat{W}$. For a given gene, the effect of genetically regulated gene expression level on the phenotype can be obtained as follows in FUSION:


(2) $$z_{g}=\frac{{\hat{W}}^{{}^{\prime }}\,Z}{\sqrt{{\hat{W}}^{{}^{\prime }}\,\text{Σ}\,\hat{W}}}$$

In comparison to individual-level data-based TWAS, GWAS summary statistics-based TWAS is more computationally efficient and has the ability to analyze a larger GWAS dataset as it is less central processing unit (CPU) and memory intensive. Various GWAS summary statistics-based TWAS methods have emerged since FUSION, including S-PrediXcan (Barbeira et al., 2018) and UTMOST (Hu et al., 2019) (Table 2).

The primary difference between TWAS that uses individual-level data and those that use GWAS summary statistics is in the estimation of LD structure for testing populations. The individual-level genotype data are usually not easily accessible from most published GWAS studies, making it difficult to examine the LD structure among eQTLs in each GWAS dataset. GWAS summary statistics-based TWAS circumvents this issue by deriving an LD matrix from a reference set, either the reference panel used for eQTL discovery, or a multi-ancestry, deeply sequenced reference panel like 1000 Genomes Project (1000 Genomes Project Consortium et al., 2015). Nevertheless, seldom does a reference population panel perfectly resemble the population structure of a specific study cohort. The discrepancy between the reference LD matrix and the actual LD structure of a study cohort will likely introduce noise and may lead to false positive or false negative results in GWAS summary statistics-based TWAS, despite a general good concordance between individual-level and summary statistics-based TWAS (Barbeira et al., 2018). The silver lining is the increasing sample sizes in reference population panels for more accurate estimates of an LD structure, which matters for GWAS summary statistics-based studies (Benner et al., 2017).

Overall, individual-level TWAS provides more accurate estimates of gene-trait associations. However, it usually takes up significant computational resources; and individual-level genotype data are not always accessible to the research community. On the other hand, GWAS summary statistics-based TWAS is advantageous in its capability to prioritize genes using only GWAS summary statistics and also computation speeds that are orders of magnitude faster than individual-level TWAS. Nevertheless, as mentioned above GWAS summary statistics-based TWAS can introduce noise to association results as the commonly used reference LD matrix cannot perfectly resemble the LD structure of the study cohort. GWAS summary statistics-based TWAS will require a greater GWAS sample size to achieve satisfactory statistical power. Because of these limitations, GWAS summary statistics-based TWAS generally needs additional validation and careful interpretation.

### eQTL Detection

The choice of eQTL database is important in TWAS (see “Statistical models for eQTL identifications” in Table 2). Quality of the eQTL databases impacts the prediction accuracy of gene expression levels. Transcriptome and genotype data of higher quality can capture greater proportions of the genetic components of gene expression regulation, identify eQTLs with moderate to small effect sizes, and improve the precision of eQTLs in complex gene regions that share the same locus control region or express multiple isoforms.

The power to detect eQTLs from transcriptome and genotype datasets is partially dependent on the sample size. Over the past decade, not only the sample sizes of reference transcriptome data, but also the diversity of human tissues and cell lines, have grown to support a deeper and broader understanding of genetic architecture of eQTLs. Better quality eQTL data in more diverse tissues have been made publicly available thanks to several consortia, including ScanDB (Gamazon et al., 2010), GTEx (GTEx Consortium, 2020), ImmVar (Ye et al., 2014), BLUEPRINT (Chen et al., 2016), CAGE (Lloyd-Jones et al., 2017), PsychENCODE (Wang D. et al., 2018), eQTLGen (Võsa et al., 2018). ScanDB is one of the earliest centralized eQTL databases that explores eQTLs in 176 HapMap Lymphoblastoid Cell Lines, made up by 87 CEPH from Utah (CEU) and 89 Yoruba from Ibadan (YRI) (Gamazon et al., 2010). Approximately five thousand eQTLs were discovered in the CEU and YRI, respectively, and are hosted on ScanDB website^1^ (Duan et al., 2008). Following ScanDB, one of the most well-known eQTL studies is the Genotype-Tissue Expression (GTEx) project that was launched in 2010 (GTEx Consortium, 2015). The latest release version of GTEx (GTEx v8) extended the search of eQTLs in 838 donors (15,201 postmortem biospecimen) for 49 primary human tissues and two cell lines (GTEx Consortium, 2020). GTEx provides tissue-specific eQTLs and splicing quantitative trait loci (sQTLs) for 18,262 protein-coding and 5,006 long intergenic non-coding RNA (lincRNA) genes after biological and statistical quality control. GTEx brings the awareness of widespread eQTL effects that almost all protein coding genes and ∼67% of lincRNA genes have been detected to be under the influence of *cis*-eQTLs in at least one tissue. An even greater eQTL detection sample size than the GTEx project has been assembled through the effort of the eQTLGen consortium^2^. eQTLGen meta-analyzed 31,684 blood samples (majority of European ancestry) from 37 datasets whose gene expression levels were profiled by three gene expression arrays and one RNA-seq platform (Võsa et al., 2018). The magnitude of the sample size allows eQTLGen to identify not only *cis*-eQTLs (within 1 Mbp to a gene), but also *trans*-eQTLs that are more than 5 Mbp away from a gene or on another chromosome. A single-cell version of eQTLGen is expected to further unravel the transcriptional regulatory mechanism behind complex disease and traits in delicate individual immune cell types (van der Wijst et al., 2020).

Interpretation of eQTL effects and TWAS results should consider the fact that transcriptional regulation is a spatiotemporal process that can differ from tissue to tissue and between life and death. Ferreira et al. (2018) found that a proportion of genes displayed drastic transcript-level changes over the postmortem intervals due to postmortem ischemia, regulatory changes, and RNA degradation. Genes that are affected by postmortem gene regulation differ from tissue to tissue (Ferreira et al., 2018). While postmortem effects on transcriptome are still largely unknown, postmortem tissues, including blood samples, remain irreplaceable natural resources to explore tissue-specific molecular mechanisms of complex diseases. Given the transcriptional regulatory difference between life and death, it is important to validate the effects of eQTLs and transcriptional changes of genes in complex trait or disease-relevant biospecimens using RNA-seq or high-throughput massively parallel reporter assay (MPRA) (Tewhey et al., 2018).

Methods to detect eQTLs are developed based on different biological hypotheses and statistical models. eQTL detection methods can differ in two parts: (1) the assumptions of the genetic architecture of transcriptional regulation, and (2) adoption of a tissue-by-tissue analytic model versus a cross-tissue method design. Due to a wider acknowledgement of the polygenic genetic architecture of intermediate molecular traits (Zhang et al., 2011; King et al., 2014), eQTL studies have set off to detect multiple potential causal eQTLs at a genetic locus, as opposed to only a single eQTL at a locus as would be done in a monogenic model. For example, Gusev et al. (2016) used Bayesian Sparse Linear Mixed Model (BSLMM) (Zhou et al., 2013) to detect eQTLs that were later used to predict gene expression levels (Table 2). BSLMM fits all SNPs nearby a gene into the model and allows two types of genetic components, one sparse (i.e., a small set of eQTLs with large effect sizes) and one vastly polygenic (i.e., all SNPs at a locus having marginal effect sizes). BSLMM attained a better prediction performance than a prediction estimated by merely using the top eQTL at a locus (Gusev et al., 2016). This suggests a non-monogenic genetic architecture of gene expression regulation, which is further supported by another contemporary study by Gamazon et al. (2015) that compared the top SNP (monogenic), polygenic score (polygenic), and elastic net (polygenic). To further understand the sparsity of polygenic genetic architecture behind gene expression, Wheeler et al. (2016) evaluated the contribution of sparse and polygenic components for transcriptional regulations, using BSLMM (sparse and polygenic), LASSO (sparse) and elastic net/ridge (polygenic) regression models. They compared the genetic heritability of gene expression explained by each method to determine the local genetic contribution of eQTLs to gene expression variation. They found that *cis* gene expression regulation was dominated by a small number of genetic variants rather than a large collection of genetic variants of marginal effect sizes. The discovery by Wheeler et al. (2016) strongly suggests a non-monogenic, sparse genetic architecture of *cis* transcriptional regulation. However, research in this area is in general impeded by limited sample sizes of transcriptome data.

Cross-tissue meta-analyses of transcriptome data have gained greater attention due to their capability of overcoming the sample size constraint as seen in the tissue-by-tissue eQTL detection approaches (Table 2). Research of cross-tissue eQTL detection is fostered by the discovery that an obvious proportion of *cis-*eQTLs are shared across all tissues and have correlated effect sizes across tissues (Battle et al., 2017). Flutre et al. (2013) introduced a cross-tissue Bayesian model that allows a proportion of eQTLs being shared across tissues and accounts for intra-individual correlations among tissues. Their hierarchical model can estimate heterogeneous effects of eQTLs in different tissues and identify eQTL active tissues. A similar approach is Meta-Tissue by Sul et al. (2013) that adopts a linear mixed model, which specifically leverages the random effects model developed by Han and Eskin (2011), to achieve similar goals as the Flutre et al. (2013). More cross-tissue eQTL detection methods have followed over years, including work by Acharya et al. (2016), RECOV by Duong et al. (2017), a sparse group LASSO model embedded in UTMOST by Hu et al. (2019), and a Joint Tissue Imputation (JTI) approach by Zhou et al. (2020). In general, cross-tissue eQTL detection methods have shown greater power in simulation studies in comparison to tissue-by-tissue approaches and a substantial increase in the numbers of identified eQTLs and eGenes (Genes that are regulated by at least one statistically significant eQTLs) (Han and Eskin, 2011; Flutre et al., 2013; Sul et al., 2013; Acharya et al., 2016; Duong et al., 2017; Hu et al., 2019; Zhou et al., 2020) (see “Statistical models for eQTL identifications” in Table 2).

### Variety of Gene-Trait Association Methods

In addition to eQTL discovery, integrative cross-tissue analyses flourish in the evaluation of TWAS gene-disease associations (Table 2). Earliest design of TWAS, i.e., PrediXcan, investigates gene-trait associations in a tissue-specific manner. Naturally, PrediXcan estimates the statistical significance of association between a disease of interest and predicted gene expression levels tissue-by-tissue. However, tissue-specific TWAS faces four issues. First, limited sample sizes of reference transcriptome data not only restrict statistical power to identify eQTLs, but also TWAS power. This can happen in a way where certain tissues do not have sufficient sample sizes and power to detect eQTLs for a functional gene. As a result, TWAS will not be able to predict the gene’s expression levels, let alone test for gene-trait associations in an underpowered tissue. Second, causal tissues of many complex diseases or traits can be unclear or unavailable, making it difficult to determine specific tissues or cell lines on which one should conduct TWAS. Third, when causal tissues are unclear, one might choose to conduct an exploratory TWAS on multiple tissues. This kind of study design invites a substantial multiple testing burden. In an exploratory situation, one will need to correct TWAS association results for 49 primary human tissues or cell lines (available by GTEx), when perhaps only one or two tissues were causal to a complex disease. On the other hand, this test-all-tissue approach also carries an implicit assumption that TWAS will only assign statistical significance to tissues that are biologically relevant to the complex trait of interest. This assumption, however, can be easily violated due to the fourth issue. Fourth, cumulative evidence has suggested that there is shared local genetic architecture of gene expression regulation and similar *cis-*eQTL effect sizes across tissues (Battle et al., 2017; Liu et al., 2017; Ongen et al., 2017). The shared eQTL effects across tissues indicates that TWAS cannot distinguish disease-relevant tissues from irrelevant tissues that share similar gene expression levels from a statistical perspective (Wainberg et al., 2019). Cross-tissue TWAS is thus promoted to resolve some of these issues with tissue-specific TWAS. Essentially, cross-tissue TWAS methods aggregate evidence across tissues to test the joint effect of gene expression levels on complex diseases or traits.

Different cross-tissue TWAS methods have been developed and provide various options for either individual-level genotype data or GWAS summary statistics (Table 2). MultiXcan by Barbeira et al. (2019) is a cross-tissue TWAS method provided within the MetaXcan method package. MultiXcan uses individual-level genotype data to predict gene expression levels in each single tissue and then fits the predictions across tissues against a phenotype in a statistical model to estimate the joint effect of a gene on a complex trait of interest. To avoid inflation of results due to correlated gene expression levels across tissues, MultiXcan adopts the principal component regression which specifically uses the first several orthogonal principal components of the predicted gene expression data matrix as explanatory variables. The GWAS summary statistics version of MultiXcan is called S-MulTiXcan (Barbeira et al., 2019). An alternative to S-MulTiXcan is a method called UTMOST developed by Hu et al. (2019) UTMOST uses a generalized Berk-Jones (GBJ) test which carries out a secondary test to examine if a gene is statistically significantly associated with a disease in at least one of the tested tissues. GBJ tests in UTMOST handles correlated gene expression levels across tissues by taking the covariance among single-tissue TWAS test statistics into account (Sun and Lin, 2020).

Cross-tissue TWAS has advantages and disadvantages in comparison to single-tissue TWAS. Cross-tissue TWAS methods have shown improved power to identify gene-level association in both simulated and natural data (Barbeira et al., 2019; Hu et al., 2019). Nevertheless, cross-tissue TWAS results are not tissue-specific and thus, cannot reveal tissue-specific genetic regulatory mechanisms. Computing resources and time required by cross-tissue TWAS methods are much higher than the corresponding single-tissue counterparts. Despite pros and cons, further validation, such as replication in independent datasets or functional validation, are needed by either single-tissue or cross-tissue TWAS.

Cross-tissue TWAS methods are not restricted to the eQTL models that come with the method. In general, a state-of-the-art eQTL method with better prediction accuracy of gene expression levels is preferred. In other words, cross-tissue TWAS methods such as MultiXcan, S-MulTiXcan (Barbeira et al., 2019) and UTMOST (Sun and Lin, 2020) can use the cross-tissue JTI-based eQTL models (Zhou et al., 2020) that is developed separately. The same principle applies to single-tissue TWAS methods. PrediXcan, S-PrediXcan and FUSION can use, for example, the cross-tissue JTI-based eQTL models which provides an improved prediction accuracy of gene expression levels (Gamazon et al., 2015; Gusev et al., 2016; Barbeira et al., 2018; Zhou et al., 2020).

## Challenges

While promising methods for disease gene discovery, TWAS faces several challenges. First, prediction accuracy of gene expression levels is limited by the heritability (h^2^) of each gene. The heritability (h^2^) of a gene’s expression levels determines the upper bound of prediction accuracy by eQTLs. On the one hand, different studies have shown that TWAS can accurately predict the expression levels for genes that are highly locally heritable (*h*^2^ ≥ 0.5) (Gamazon et al., 2015; Li et al., 2018). And 59% of genes in the DGN whole blood have well estimated local h^2^ (FDR < 0.1) (Wheeler et al., 2016). On the other hand, some genes have little to negligible estimated local heritability and should be removed from TWAS to avoid false positives. Nonetheless, much is still unclear about the heritability of gene expression levels across tissues and beyond *cis-*eQTLs.

Thus far, TWAS has only been using *cis-*eQTLs within a certain distance from genes. This is consistent with observations in several studies that the majority of *cis*-eQTLs cluster around the transcription start site of the target gene (Nica et al., 2011; GTEx Consortium, 2015). However, gene can be regulated by both *cis* and *trans*-regulatory elements in the human genome. Many studies seek to identify *trans*-eQTLs, which have been absent in gene expression heritability estimation due to technical limitations. Several previous studies estimated that ∼70% of the genetic heritability of gene expression levels could be attributable to *trans*-eQTLs that are on another chromosome or more than 5 Mb away (Boyle et al., 2017; Liu et al., 2019), indicating the importance of *trans-*eQTLs in transcriptional regulation. However, *trans*-eQTL studies face enormous multiple testing burden. Studies to identify *trans*-eQTLs will need to test all possible intra and inter-chromosome variant-gene pairs. The total number of statistical tests is orders of magnitude greater than that of *cis-*eQTLs, which only considers proximal variant-gene pairs. A great number of samples is thus needed for *trans*-eQTL research to guarantee sufficient statistical power (Westra et al., 2013). Even if *trans*-eQTL data are made available, as in blood-related cell lines by eQTLGen (Võsa et al., 2018), TWAS may still have difficulty utilizing *trans*-eQTLs due to two key factors. First is the possible overlapping effects between the *trans* and *cis*-eQTLs for a target gene. *Trans*-eQTLs likely regulate expression of a *trans*-acting TF, which subsequently functions by binding to a *cis*-regulatory element where a *cis*-eQTL resides (Võsa et al., 2018). Second is the difficulty of calculating LD among eQTLs. The computing time and resources needed for such a task are exponentially greater than that for *cis-*eQTLs.

Another challenge in TWAS is the lack of eQTL data from different ancestry groups, diseases, medical conditions, sex, etc. The majority of samples used for large-scale eQTL studies were of European ancestry. eQTL databases that were prepared by a few earlier TWAS methods were exclusively European ancestry individuals (Gamazon et al., 2015; Gusev et al., 2016; Barbeira et al., 2018). Ancestry-specific eQTL data are available for some ancestry groups, but these resources are generally limited. The Multi-Ethnic Study of Atherosclerosis (MESA) characterized eQTLs in African American (*N* = 233), Hispanic (*N* = 352), and European (*N* = 578) populations, separately (Mogil et al., 2018). However, the MESA genotype and RNA-seq data were collected from only CD14+ monocytes and individuals free of clinical cardiovascular diseases (CVD) at recruitment. Although, individuals with CVD and other medical conditions are likely to experience different transcriptional regulation from their healthy peers. Overall, much is still to explore about the eQTLs in different ancestries, medical conditions, age, sex, etc. (Piasecka et al., 2018).

It is hard to quantify TWAS power due to the complexity of transcriptional regulation and varied genetic backgrounds of different complex diseases or traits (Veturi and Ritchie, 2018; Li et al., 2021). For example, TWAS power can be influenced by the quality of gene expression prediction (sample sizes used for eQTL detection, concordance between transcriptome reference population and testing populations, coverage of eQTLs in the test dataset, etc.), or genetic factors (e.g., genetic heritability of gene expression levels, heritability of the phenotype, sample size, MAF, etc.). On top of the aforementioned factors, TWAS is also challenged by the fact that causal tissues or cell types are unclear in the majority of complex diseases or traits. Overall, TWAS statistical power is contingent on so many varied factors that it is hard to estimate TWAS power without making a delicate set of assumptions; and one should be careful when interpreting TWAS power.

Transcriptome-wide association studies need fine-mapping. Statistically significant TWAS results indicate only association, but not causation. Statistically significant genes are likely tag genes for other causal genes in its proximity, but achieve the greatest statistical significance due to various reasons (Wainberg et al., 2019). One solution is to fine-map causal genes by leveraging the LD structure among genes. For example, the method FOCUS estimates a set of credible genes that are tagged by a statistically significant gene by analyzing the patterns of eQTLs, GWAS signals and surrounding LD structure (Mancuso et al., 2019). One will have certain degree of statistical confidence (90 or 95% by choice) that causal genes are within the set of credible genes. The fine-mapping capability of FOCUS was supported by its success in recovering *SORT1* gene as one of the LDL risk genes. More work is expected in this field of research (Mancuso et al., 2019; Wu and Pan, 2020).

## Future Directions

Understanding the genetic architecture of complex diseases and traits is still an ongoing task for the field of translational medicine. The journey from bench science to bed-side care requires the knowledge of causal genes, pathways, and mechanisms behind complex traits. The cumulative number of non-coding GWAS discoveries, time and again, stresses the need to fill the gap between non-coding genetic variants and downstream affected genes in order to uncover complex trait mechanisms. In this review, we categorize two types of methods that integrate GWAS with functional genomics data to bridge the variant-to-gene gap – fine-mapping approaches and gene prioritization approaches. We discuss the background, pros and cons of several classes of developed TWAS methods, influential factors in TWAS analyses, and challenges.

We expect greater endeavors in TWAS and functional genomic studies for a variety of geographical ancestry groups in the next 10 years, including but not limited to African, Asian, Hispanic or Latin, Greater Middle Eastern, Native American, Oceanian, and admixed populations (Lavange et al., 2010; H3Africa Consortium et al., 2014; Kowalski et al., 2019; Choudhury et al., 2020; Gay et al., 2020; Shang et al., 2020). Generation of these eQTL data will require resources and efforts from the research communities in different parts of the world.

High-throughput next-generation sequencing technology and array-based platforms will continue to generate informative functional genomics data. Ripening 3C and 3C-derived technologies will generate more knowledge about chromatin loop-assisted *cis* and *trans* regulatory interactions. Increasing evidence suggests the prevalence of distal regulatory mechanisms that cannot be easily captured with local LD structure (Whalen and Pollard, 2019; GTEx Consortium, 2020). Mumbach et al. (2017) recently developed HiChIP that generates high-resolution contact maps for enhancer-promoter interactions in a human coronary artery disease-related (CAD-related) cell type. They found that ∼89% of the coronary artery disease-associated SNPs skipped at least one gene to reach predicted target genes. The extent to which distal transcriptional regulation occurs is still unknown in the majority of complex human diseases or traits. But genomic regulatory information will be useful to decipher functionality of non-coding variants and map non-coding variants to their downstream affected genes.

Another highly expected sequencing technology by the field of eQTL and TWAS studies is the single-cell RNA sequencing (scRNA-seq) (Tang et al., 2009). Bulk RNA-seq of a tissue sample is the most economical way to obtaining transcriptome data in a large scale, despite the fact that a tissue sample comprises more than one cell type. Different cell types undergo distinguished genetic regulation that makes up their specific cellular identities. A gene’s expression levels in a tissue, thus, are likely to differ from a cell type to another cell type. scRNA-seq profiles cell type composition in a tissue at a refined resolution and allows exploration of transcriptome heterogeneity across cell types (Snijder and Pelkmans, 2011). Growing scRNA-seq data and analytic methods will pave a new avenue in eQTL research that performs eQTL studies in various cell types in a tissue (van der Wijst et al., 2018). This will improve precision and accuracy of eQTLs. On the other hand, having a grasp on which causal tissues or cell types are important for a given complex disease will be essential for developing a better understanding of disease mechanism and clinical treatment. scRNA-seq data promise greater statistical power to identify complex trait-relevant tissues or cell types by providing distinguishable transcriptome profiles among cell types (Ongen et al., 2017; Finucane et al., 2018). Several scientific consortia have initiated the effort in generating scRNA-seq data in large sample sizes and multiple tissues, including the Human Cell Atlas (Regev et al., 2017), Single-cell eQTLGen (van der Wijst et al., 2020) and the LifeTime consortium (Rajewsky et al., 2020). At this dawn of single-cell omics sequencing technology, sample sizes and diversity of tissues and cell types will likely continue to be limited.

Even though genes are considered functional and heritable units, there is a shortage of gene-centric functional annotation models. Existing functional annotation models focus on generating regulatory hypotheses for non-coding variants on a variant-centric basis. For most genes, it is unclear how the gene is regulated by different genetic regulatory elements, despite the fact that an average of 3.9 distal elements interact with the transcription start site (TSS) of a gene (Sanyal et al., 2012). The shortage of gene-centric functional annotation models also prevents locus-based statistical methods from combining *cis* and *trans*-regulation. With the advances in sequencing technologies, we are expecting a better understanding of genomic regulation that incorporates *cis* and *trans*-regulation to investigate how dysregulation of a gene, as a functional unit, contributes to complex diseases or traits.

More than a decade into GWAS research of complex disease, the molecular mechanisms behind most complex diseases remains unclear due to the valley between non-coding GWAS signals and the downstream affected genes. The next two decades await more research that sheds new light on complex disease mechanisms to promote novel therapeutics and precision medicine.

## Author Contributions

BL conceived the idea, drafted, and revised the manuscript. MR extensively revised the manuscript. Both authors listed have made a substantial, direct and intellectual contributions to the work, and approved it for publication.

## Conflict of Interest

The authors declare that the research was conducted in the absence of any commercial or financial relationships that could be construed as a potential conflict of interest.

## Publisher’s Note

All claims expressed in this article are solely those of the authors and do not necessarily represent those of their affiliated organizations, or those of the publisher, the editors and the reviewers. Any product that may be evaluated in this article, or claim that may be made by its manufacturer, is not guaranteed or endorsed by the publisher.

## Footnotes

1
http://www.scandb.org/

2
https://www.eqtlgen.org/
